# RGB-D based multi-modal deep learning for spacecraft and debris recognition

**DOI:** 10.1038/s41598-022-07846-5

**Published:** 2022-03-10

**Authors:** Nouar AlDahoul, Hezerul Abdul Karim, Mhd Adel Momo

**Affiliations:** 1 Faculty of Engineering, Multimedia University, Cyberjaya, Malaysia; 2 YO-VIVO Corporation, Bacolod, Philippines

**Keywords:** Computational science, Computer science

## Abstract

Recognition of space objects including spacecraft and debris is one of the main components in the space situational awareness (SSA) system. Various tasks such as satellite formation, on-orbit servicing, and active debris removal require object recognition to be done perfectly. The recognition task in actual space imagery is highly complex because the sensing conditions are largely diverse. The conditions include various backgrounds affected by noise, several orbital scenarios, high contrast, low signal-to-noise ratio, and various object sizes. To address the problem of space recognition, this paper proposes a multi-modal learning solution using various deep learning models. To extract features from RGB images that have spacecraft and debris, various convolutional neural network (CNN) based models such as ResNet, EfficientNet, and DenseNet were explored. Furthermore, RGB based vision transformer was demonstrated. Additionally, End-to-End CNN was used for classification of depth images. The final decision of the proposed solution combines the two decisions from RGB based and Depth-based models. The experiments were carried out using a novel dataset called SPARK which was generated under a realistic space simulation environment. The dataset includes various images with eleven categories, and it is divided into 150 k of RGB images and 150 k of depth images. The proposed combination of RGB based vision transformer and Depth-based End-to-End CNN showed higher performance and better results in terms of accuracy (85%), precision (86%), recall (85%), and F1 score (84%). Therefore, the proposed multi-modal deep learning is a good feasible solution to be utilized in real tasks of SSA system.

## Introduction

The activity program of space agencies (European Space Agency, National Aeronautics and Space Administration) includes launching a new satellite, navigating the solar system, and forecasting the earth climate. In the last decades, a huge amount of space debris has been generated by the space agencies which orbit the earth, and thus there is a big need for space situational awareness (SSA) program. This SSA program which acts as an alarm system in solar system^1^ was found to address the increasing number of the space debris. SSA has three main functions including space surveillance and tracking to track satellite and space debris, weather detection and forecasting, and detection of space objects such as debris to reduce their effects on the earth^1^.

The space object recognition is a significant task in space missions to classify various objects such as spacecrafts and debris. The difficulty of recognition task was caused by lack of visual data used for training the classification model. The process of data collection is complex and costly. Therefore, synthetic data generation under a photo-realistic space simulation environment were found to address the previous problem^2^. The data should be largely diverse with extreme and challenging sensing conditions. A novel dataset called SPAcecraft Recognition leveraging Knowledge of space environment (SPARK) was found and shared with the research community in ICIP 2021 challenge^2,3^.

The approach of deep learning which is data hungry requires large number of samples to train the model. In this paper, SPARK dataset^2^ has ten thousand of images used for training the proposed solution of multi-modal learning. The objective was to classify the space objects such as spacecraft and debris into eleven categories including AcrimSat, Aquarius, Aura, Calipso, Cloudsat, CubeSat, Debris, Jason, Sentinel-6, Terra, and TRMM.

A deep learning model called vision transformer was proposed by Vaswani et al.^4^ for natural language processing (NLP) tasks especially for machine translation. The transformer was transferred to computer vision tasks such as image classification inspired by the successes of the transformer in NLP. The outcome was a vision transformer that was found to outperform CNN-based methods in various applications including image recognition of small or mid-sized images such as ImageNet, CIFAR-100, VTAB^5^, object detection^6,7^, image segmentation^6,8^, image generation using transformers-based GAN^6,9^, image colorization^6,10^, clustering^6,11^, and 3D analysis^6,12^. To the best of our knowledge, this is the first paper that targets using the vision transformer for space object classification utilizing SPARK dataset proposed by ICIP2021 challenge organizers^2,3^.

This paper highlights an interesting challenge for the research community. It contributes to the body of knowledge as follows:

1. A space object classification model is proposed to classify objects into debris and spacecraft. Additionally, it distinguishes between various categories of spacecrafts.
2. A novel multi-modal learning is proposed for spacecraft classification utilizing a combination of vision transformer using RGB images and End-to-End CNN using depth images.
3. The proposed multi-modal learning was evaluated and compared with existing CNN based methods such as ResNet50, EfficientNetB7, and DenseNet201.
4. The concept of domain generalization from natural images to the space imagery domain was explored to transfer deep representation from ImageNet to SPARK images.
5. An ablation study was done to validate the significance of multi-modal learning over single learning that uses RGB images only. Average decision approach was added to combine the two decisions made from two models into one final decision. This leads to an increase in the classification accuracy.

This paper is organized as follows: "Related work" describes the SPARK space imagery dataset. Additionally, it demonstrates the approach of transfer learning, End-to-End CNN, pre-trained deep CNNs, pre-trained vision transformer, and multi-modal learning. In "Materials and methods", the experiments and results are discussed in detail. Furthermore, the comparison between the proposed solution and existing methods is done. Finally, "Results and discussion" summarizes the outcome of this work and gives readers a glimpse into potential improvements in the future.

## Related work

The task of target recognition should be done autonomously to minimize the risk of collision in space^13^. The vision-based sensor such as camera^2,14–16^ is the most significant component in SSA to observe visual data and build data-driven AI solution. Various methods have been proposed in previous research works to track and monitor inactive and active satellites from one side and remove space debris from the other side. LiDAR sensor was also used for debris removal, target detection, and pose estimation^2,15–17^. Pose estimation methods were found to match 3D spacecraft wireframe (target) with 2D image utilizing the matching process between visual features extracted from both image and wireframe^18^. The Perspective-n-Point (PnP) problem was solved to find the pose^18^. The conventional computer vision algorithms such as Sobel and Canny detectors were used to extract the edge features^19,20^. On the other hand, traditional machine learning algorithms were considered in the task of pose estimation utilizing principal component analysis (PCA)^21^. The PCA was applied to a query spacecraft image and then compared with the ground truth poses in the dataset for matching purposes.

Object detection and image classification are two main tasks in computer vision to detect the objects, calculate their bounding boxes, and predict the categories. Deep learning algorithms have produced better results than computer vision algorithms because they use automatic feature learning and extraction. Therefore, deep learning algorithms have been used in the space applications to recognize spacecraft and debris for various purposes. Pre-trained convolutional neural network was one of the deep learning models used to estimate the pose of the spacecraft^22,23^ such as GoogLeNet CNN^24,25^. On the other hand, to determine the translation, and rotation of a space object relative to a camera, VGG CNN^26,27^ was trained and tested on synthetic dataset. Furthermore, to estimate the pose of uncooperative spacecraft without 3D information and to predict the bounding box of space objects, ResNet CNN was demonstrated^18,28^.

The performance of deep learning and its generalization ability are based on the size of data fed to deep model. The data size should be large to produce the expected improvement compared to traditional machine learning methods. In space application, the cost of spacecraft data acquisition is expensive. Therefore, various synthetic datasets were proposed in research works for 6D pose estimation including Unreal Rendered Spacecraft On-Orbit (URSO) dataset^29^ and Spacecraft pose estimation dataset (SPEED)^30,31^.

In addition to the cost of space data acquisition, object tracking is a complex task because the surrounding spacecrafts or targets are varied in sizes. To address the previous problems, researchers have considered the data acquisition process to collect images of space objects such as spacecraft and debris. Therefore, they generated high resolution synthetic spacecraft dataset using Unity3D game engine environment simulator^32^. To propose sufficient labelled space dataset, a novel SPARK dataset was found specifically for space object classification^2,3^. The SPARK dataset was represented by realistic earth, and the surrounding objects around the earth. ResNet^28^ and EfficientNet^33^ were demonstrated as pre-trained CNNs utilizing SPARK dataset with several scenarios^2^. The three scenarios are: (1) random initialization of the models and training from scratch. (2) feature extraction by freezing the backbone and training only the classifier in top layers. (3) using the pre-trained weights and then fine-tuning the whole model including the backbone and classifier. They found that the models trained on both RGB, and depth images showed better performance than single models^2^.

## Materials and methods

This section describes the dataset used in this work to shed light on the challenging contents available in the images. Additionally, the approach of transfer learning is demonstrated using CNN based models such as ResNet50, EfficientNetB7, and DenseNet201 and state-of-the-art vision transformer. Furthermore, the multi-modal learning is discussed. Finally, the model’s architectures and hyperparameters are described in detail.

### Datasets overview

**Figure 1:**
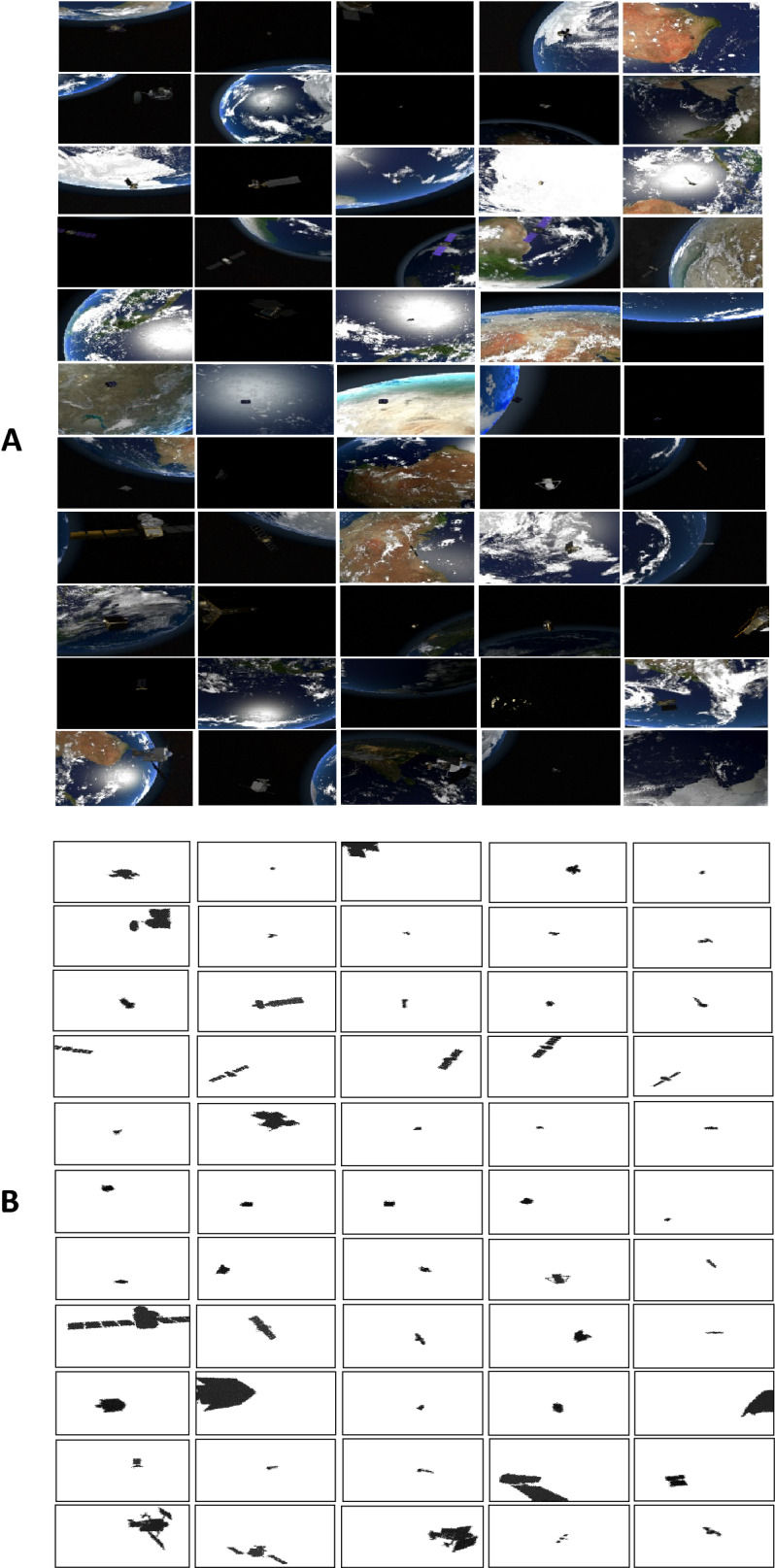
(**A**) Few samples of RGB images with various object sizes and backgrounds from the spark dataset^2,3^ including AcrimSat, Aquarius, Aura, Calipso, Cloudsat, CubeSat, Debris, Jason, Sentinel-6, Terra, and TRMM in the rows 1,2,3,4,5,6,7,8,9,10, and 11 respectively. (**B**) Few samples of corresponding Depth images.

This paper demonstrates a novel space dataset called SPAcecraft Recognition leveraging Knowledge (SPARK) of space environment that was proposed in ICIP 2021 challenge^2,3^. A total of 150 k of RGB images and another 150 k of depth images were generated from Unity3D game engine environment simulator. The proposed dataset was utilized for space object classification into eleven categories including one debris and 10 satellites^2,3^. Five classes of debris were divided into 5 k images for each debris class. The five classes of debris were grouped into one set called debris category with 25 k images. On the other hand, ten categories of satellites with 12.5 K images for each include AcrimSat, Aquarius, Aura, Calipso, CloudSat, CubeSat, Jason, Sentinel-6, Terra, and TRMM. The space objects were acquired from NASA 3D resources^2,34^. Figure 1 shows several RGB images and their corresponding depth images from the SPARK dataset.

The 150 k of images in SPARK dataset were divided into 60% (90,000 images), 20% (30,000 images), and 20% (30,000 images) for training, validation, and testing, respectively^2,3^. While RGB images have resolution of 1024 × 1024, depth images have resolution of 256 × 256. Only labels of training and validation images were given. Therefore, validation set was used as testing set. Additionally, training set was divided into training and validation sets. Various challenges are available in this dataset, and they are summarized as follows^2^:

1. the target locations are distributed randomly in the field of view of a camera which was mounted on a chaser. Additionally, the chaser model has several orientations and ranges. Furthermore, Earth and Sun are rotated randomly around their axes.
2. high contrast images with lighting changes. The models of Sun flares, rays, and reflections from the space to Earth were designed and built.
3. various contents of backgrounds with different orbital scenarios including black background, sparsely illuminated stars in the background, Earth with oceans and clouds, and object with night side or day side of Earth in the background.
4. highly noisy spaceborne images with small size of sensor and highly dynamic range imaging.
5. various object sizes with several ranges and distances between the target spacecraft and the camera attached to the chaser.

### The proposed solution

This section aims to demonstrate the proposed solution for classification of space objects including spacecraft and debris. The architecture and hyperparameters of proposed supervised End-to-End CNN are described. Additionally, the architectures of deep CNNs such as ResNet50, EfficientNetB7, and DenseNet201 are demonstrated to transfer learning between various domains. Furthermore, the architecture and hyperparameters of vision transformer are explored. Finally, the approach of multi-modal learning is described in detail.

#### Classification of depth images with End-to End CNN

Deep neural networks are special type of neural networks with larger number of layers. They were used with big data to enhance the model performance in terms of accuracy in various applications such as human activity recognition^35^, distortion classification^36^, and pornography detection^37^. Convolutional neural network (CNN) was found to capture spatial correlations better than fully connected layers of deep neural network (DNN) and thus it can give better performance in tasks of image classification^38^. In this paper, the End-to-End CNN was utilized for supervised feature learning to learn features from depth images. The features were mapped to eleven categories.

For end-to-end CNN training, the images were resized to 224 × 224 and rescaled by dividing the pixels by 255.Various architectures were evaluated to select the optimal one with the best accuracy. Tables 1 and 2 show the optimal architecture and hyperparameters of End-to-End CNN, respectively. The training set was divided into two sets by 80/20 rule: 80% for training and 20% for validation (tuning hyperparameters and optimize the architectures).

**Table 1:** End-to-End CNN architecture.

Layer number	Layer type
1	Input Layer with 224 × 224 × 3 image resolution
2	Conv2D with 32 3 × 3 filters
3	ReLU activation
4	Conv2D with 64 3 × 3 filters
5	ReLU activation
6	Maxpooling with 3 × 3 pool size
7	Conv2D with 128 3 × 3 filters
8	ReLU activation
9	Maxpooling with 3 × 3 pool size
10	Conv2D with 256 3 × 3 filters
11	ReLU activation
12	Maxpooling with 3 × 3 pool size
13	Flatten Layer
14	Fully connected layers with 512 nodes
15	ReLU activation
16	Fully connected layers with 11 nodes
17	Softmax activation

**Table 2:** End-to-End CNN hyperparameters.

Hyperparameter	Value
Loss Function	Categorical Crossentropy
Optimizer	Adam
Reduce_Learning_Rate_OnPlateau	Factor = 0.2, min_LR = 1e-6, patience = 2
Batch Size	32
Epochs	50

#### Transfer learning with residual network

The gradient vanishing and low accuracy are a common problem that several very deep CNNs suffer from^28^. Residual Network (ResNet) which is a very deep CNN was found to address this problem utilizing skip connections^28^. It consists of millions trainable parameters and a large number of layers such as 50, 101, and 152 layers. ResNet50 has lower number of layers with very high generalization to extract very informative features from images that have not been trained on. Therefore, it has been used to transfer learning between various domains. To achieve the optimal performance, ResNet50^28^ was trained on a large-scale dataset such as ImageNet^39^ that includes 1000 classes and 1.3 M images. After training, ResNet50 was used as a pre-trained CNN to extract features from novel small or medium-scale datasets such as SPARK^2^. In this paper, ResNet50 was used to extract 2048 features from RGB images resized to 224 × 224 in SPARK dataset. Support vector machine (SVM)^40^ was added as a replacement of top layers to map features extracted from RGB images to eleven categories.

#### Transfer learning with EfficientNet network

To scale models, an arbitrarily increasing of network depth and width or applying larger resolution of input images are performed. However, these methods require manual tuning to enhance the accuracy. Therefore, EfficientNet was found to achieve the higher accuracy on ImageNet and faster inference than the existing CNNs^33^ by balancing width, depth, and resolution. Various scaling architectures of EfficientNet such as B0, B1, B2, B3, B4, B5, B6, B7 have been demonstrated. Compared to others, EfficientNetB7 was able to improve the accuracy largely with the cost of more FLOPS. In this paper, EfficieneNetB7 has been utilized to transfer learning from ImageNet domain^39^ to space domain^2^. It was used to extract 2560 features from RGB images resized to 600 × 600 in SPARK dataset. Support vector machine (SVM)^40^ was used to replace top layers to map RGB features to eleven categories.

#### Transfer learning with DenseNet network

Dense Convolutional Network (DenseNet) introduced direct connections between any two layers with the same feature-map size^41^. It is less prone to overfitting and improved the accuracy with less computation. To achieve the optimal performance, DenseNet^41^ was trained on ImageNet^39^. In this paper, DenseNet201 has been utilized because it can balance between low error and low parameters and FLOPs. It was able to transfer learning from ImageNet domain to space domain. 1920 features were extracted by DenseNet201 from RGB images resized to 224 × 224. Support vector machine (SVM)^40^ was also utilized instead of top layers to map RGB features to eleven categories.

#### Transfer learning with vision transformer

Inspired by Dosovitskiy et al.^5^, state-of-the-art deep learning model called vision transformer was proposed for image classification in various tasks. The architecture of vision transformer is very similar to language Transformer. In other words, a sequence of 2D patches is flattened in a sequence of vectors $x \in {\mathbb{R}}^{N \times ({P}^{2}. C)}$ instead of a 1D sequence of language embeddings. The image is divided into $N=\frac{\left(H\times W\right)}{{P}^{2}}$ number of patches with patch size $\left(P, P\right).$ The patches are mapped to latent vectors with hidden size $D=768$. The output of this projection is called patch embeddings. To capture the order of patches and produce a correct sequence of vectors, position embeddings ${E}_{pos}$ are added to patches. An extra learnable “classification token” ${z}_{0}^{0}= {x}_{class}$ is added to the sequence of embedded patches for classification purposes. The vectors are applied to input of transformer encoder. The output of the transformer’s encoder $({z}_{L}^{0})$ represents the image^5^.

1 $${z}_{0}={[x}_{class}; {x}_{p}^{1} E; {x}_{p}^{2} E . . . ; {x}_{p}^{N} E]+ {E}_{pos}, {E}_{x} \in {\mathbb{R}}^{\left({P}^{2}. C\right) \times D}, {E}_{pos} \in {\mathbb{R}}^{\left(N+1\right) \times D}$$

where $P=16$, W is the image width, $H$ is the image height, $C$ is the number of channels.

**Figure 2:**
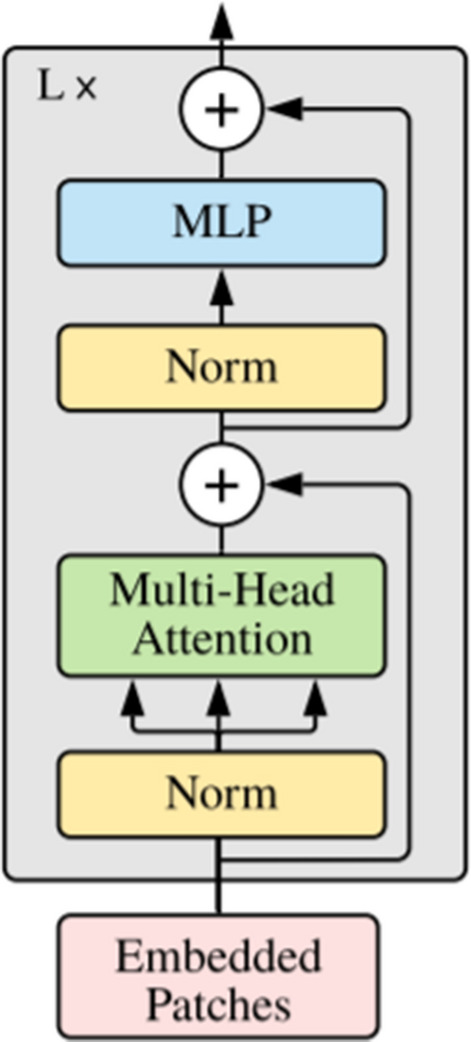
Encoder Architecture^5^.

Figure 2 shows the architecture of transformer encoder with $L$ blocks. Every block includes alternating layers of multi-head self-attention^4^ and multi-layer perceptron blocks. The layer normalization^42^ was added before every block, and residual connections were added after every block^5^.

2 $${z}_{l}^{{\prime}}=MSA\left(LN\left({z}_{l-1}\right)\right)+{z}_{l-1}$$

3 $${z}_{l}=MLP\left(LN\left({z}_{l}^{{\prime}}\right)\right)+{z}_{l}^{{\prime}}$$

4 $$y=LN({z}_{L}^{0})$$

where $$l=1\dots L.$$

**Figure 3:**
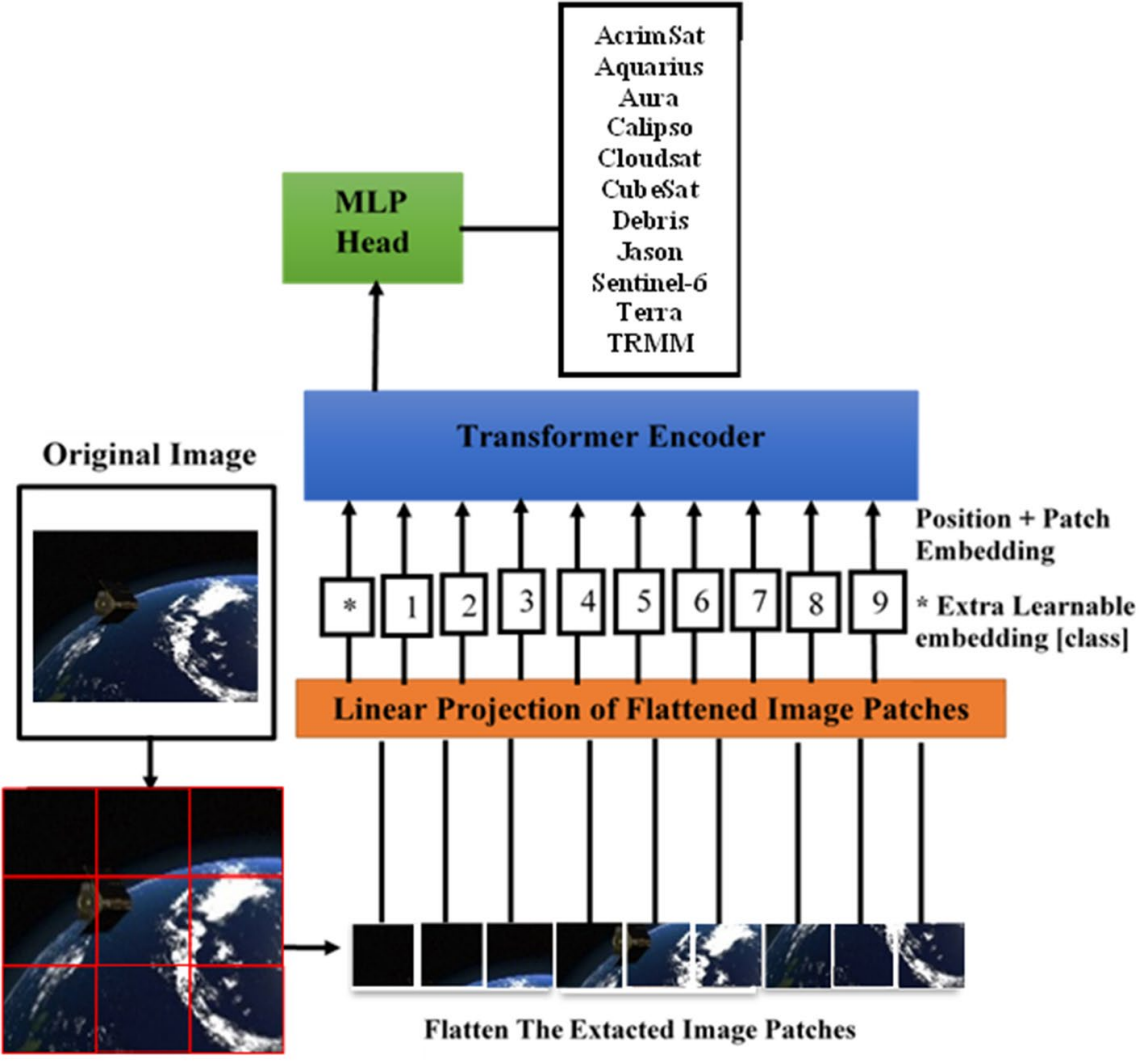
The vision transformer architecture^5^.

Usually, three datasets including ILSVRC-2012 ImageNet (1000 classes and 1.3 M images) and ImageNet-21 k (21 k classes and 14 M images)^39^, and JFT (18 k classes and 303 M images)^43^ were used to train the vision transformer model. After that, it was fine-tuned on new target small or medium-scale dataset. In this paper, the transformer pre-trained on imagenet21k and fine-tuned on imagenet2012 was used to transfer learning and representation to space images that have space object such as spacecraft or debris. A space image consists of a sequence of patches encoded as a set of words and applied to the encoder as shown in Fig. 3.

There are three types of models in vision transformer including Base, Large, and Huge^5^. The vision transformer model used in this paper is Base model which has 12 layers with 768 hidden size, 3072 MLP size, and 12 heads. The total number of parameters in this model is 86 M. The architecture and hyperparameters of vision transformer are shown in Tables 3 and 4, respectively.

**Table 3:** Vision Transformer (vit) architecture.

Layer number	Layer type
1	vit_model with 12 layers
2	Fully connected layers with 512 nodes
3	ReLU activation
4	Fully connected layers with 512 nodes
5	ReLU activation
6	Fully connected layers with 11 nodes
7	Softmax activation

**Table 4:** Vision Transformer hyperparameters.

Hyperparameter	Value
Loss Function	Categorical Crossentropy
Optimizer	Adam
Learning_rate	1e-3
Reduce_Learning_Rate_OnPlateau	Factor = 0.1, min_delta = 1e-4, patience = 2
Batch Size	128
Epochs	15

After training and fine-tuning, vision transformer was used as a pre-trained model to extract features from novel small or medium-scale datasets such as SPARK^2^. In this paper, vision transformer was used to extract 768 features from RGB image. The images in SPARK dataset were resized to 384 × 384 pixels. Three fully connected layers were added as shown in Table 3 as a replacement of top layers to map features extracted from RGB images to eleven categories.

#### The proposed multi-modal learning

**Figure 4:**
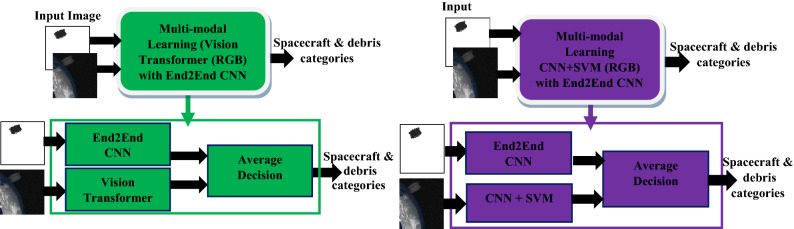
Illustration of the proposed multi-modal learning which combines vision transformer with End2End CNN (on the left) and multi-modal learning which combines CNN + SVM with End2End CNN (on the right) for spacecraft and debris classification.

The proposed multi-modal learning was done to classify various space objects such as spacecraft and debris. The proposed solution consists of two models. The first model is vision transformer pre-trained on ImageNet 21 k dataset and fine-tuned on ImageNet 2012. The transformer was used for feature extraction only without being fine-tuned with space images. Only top layers of transformer were tuned with space RGB images to produce eleven categories. The second model is End-to-End CNN used to learn features from depth images and map them to eleven categories. The proposed solution combines the previous two models (vision transformer and End-to-End CNN) to make the final decision. The average decision block was added to make the final decision regarding the final category. The block diagram of the proposed solution is shown in Fig. 4.

The proposed solution was compared with various multi-modal learning methods that used only CNNS. The methods consist of two models. The first model is deep CNN such as ResNet50, EfficientNetB7, or DenseNet201 pre-trained on ImageNet 2012. The pre-trained CNNs were used for feature extraction by freezing the backbone and adding by support vector machine (SVM) classifier instead of top layers to be trained with space RGB images to produce eleven categories. The second model is End-to-End CNN used to learn features from depth images and map them to eleven categories. The average decision block was added to make the final decision regarding the final category. The block diagram of CNN based multi-modal learning methods is shown in Fig. 4.

## Results and discussion

### Experimental setup

The training, evaluation, and testing for the proposed multi-modal learning were conducted using TensorFlow and Keras-vit frameworks and libraries. The ResNet50, DenseNet201, and EfficientNetB7 were implemented on a NVIDIA GeForce GTX 1080 Ti GPU with 64 GB RAM and 12 GB GPU RAM. On the other hand, vision transformer was trained on 4 × Nvidia Tesla V100 with 64 GB GPU RAM and 90 GB RAM. RGB input images were resized to 224 × 224 pixels in ResNet50, and DenseNet201. On the other hand, they were resized to 600 × 600 and 384 × 384 in EfficientNetB7, and vision transformer, respectively. The depth input images were resized to 224 × 224 and applied to each End-to-End CNN. For SVM, various values of regularization parameters C and Kernel functions f were evaluated to find the best accuracy. The optimal values were C = 50, and f = polynomial.

### Experimental results

In this section, the performance of proposed solution of multi-modal learning that combines End-to-End CNN and vision transformer is evaluated. Moreover, the proposed solution is compared with other CNN based multi-modal learning such as ResNet50, DenseNet201, and EfficientNetB7 combined to End-to-End CNN.

To evaluate the classification performance, several performance metrics such as accuracy, precision, recall, and F1 score were utilized. This section describes the performance metrics as follows:

1. Accuracy is a measure that calculates number of samples predicted correctly over all available samples.

5 $$\mathrm{Accuracy }=\frac{TP+TN}{TP+TN+FP+FN}$$

2. Recall (Sensitivity) is a measure that calculates the proportion of actual positives that are identified correctly

6 $$\mathrm{Recall }= \frac{TP}{TP+FN}$$

3. Precision (positive predictive value) is a measure that calculates the proportion of positive identifications that are actually correct

7 $$\mathrm{Precision}=\frac{TP}{TP+FP}$$

where TP: True Positive, TN: True Negative, FP: False Positive, FN: False Negative.

4. F1 score: This metric summarizes recall and precision in one term.

8 $$\mathrm{F}1\mathrm{ score }= \frac{2 \times precision \times recall}{precision+recall}$$

The first experiment was conducted to compare between End-to-End CNN and a pre-trained DenseNet201 CNN + SVM models used for classification of depth images that have space objects. Table 5 shows recall, precision, and F1 score of End-to-End CNN for each class of eleven classes. The average of accuracy, recall, precision, and F1 score were 70%, 70%, 69%, and 69%, respectively. End-to-End CNN was able to classify Cloudsat category with F1 score of 52%. Additionally, Table 6 shows recall, precision, and F1 score of a pre-trained DenseNet201 CNN + SVM for each class of eleven classes. The average of accuracy, recall, precision, and F1 score were 68%, 67%, 68%, and 68%, respectively.

**Table 5:** Recall, precision, and F1-score of the End-to-End CNN with depth images.

Category	Precision	Recall	F1-score
AcrimSat	0.69	0.82	0.75
Aquarius	0.66	0.74	0.70
Aura	0.87	0.77	0.81
Calipso	0.59	0.51	0.55
Cloudsat	0.57	0.47	0.52
CubeSat	0.87	0.92	0.90
Debris	0.68	0.69	0.69
Jason	0.57	0.51	0.53
Sentinel-6	0.72	0.81	0.76
Terra	0.57	0.56	0.56
TRMM	0.84	0.87	0.86
Average	0.69	0.70	0.69

**Table 6:** Recall, precision, and F1-score of the DenseNet201 CNN + SVM with depth images.

Category	Precision	Recall	F1-score
AcrimSat	0.62	0.70	0.66
Aquarius	0.66	0.70	0.68
Aura	0.74	0.74	0.74
Calipso	0.65	0.59	0.62
Cloudsat	0.52	0.47	0.50
CubeSat	0.85	0.86	0.85
Debris	0.63	0.71	0.67
Jason	0.62	0.53	0.57
Sentinel-6	0.66	0.72	0.69
Terra	0.65	0.55	0.60
TRMM	0.89	0.84	0.87
Average	0.68	0.67	0.68

It is obvious that End-to-End CNN outperformed a pre-trained DenseNet201 CNN that was trained on ImageNet by 2% of accuracy and 1% of F1 score using depth images. Therefore, End-to-End CNN was used to classify depth images in all experiments related to multi-modal learning.

The second experiment was carried out to compare between various pre-trained CNN models. The performance of three pre-trained CNNs including ResNet50, DenseNet201, and EfficientNetB7 combined to SVM was evaluated for classification of RGB images that have space objects. Both ResNet and EfficientNet were utilized in^2^ for space domain in three scenarios. They used small versions of these two models to reduce computation. We implemented larger versions with the scenario of feature extraction by freezing the backbone and training only the classifier in the top layers to compare with our proposed method. This scenario was selected because the proposed solution of vision transformer was also used to extract features without tuning the backbone parameters.

Table 7 demonstrates recall, precision, and F1-score of the multi-modal learning that combines ResNet50-SVM using RGB images and End-to-End CNN using depth images. The features extracted from RGB images in ResNet50 CNN were not discriminative to recognize Cloudsat spacecraft. Therefore, the F1 score of this category is so low (6%). On the other hand, End-to-End CNN was able to recognize features of Cloudsat with 52% F1 score. Therefore, the F1 score of this category was increased to 48% in multi-modal learning method. The average of recall, precision, and F1 score were 79%, 81%, and 79%, respectively as shown in Table 7.

**Table 7:** Recall, precision, and F1-score of the multi-modal ResNet50 + End-to-End CNN.

Category	Precision	Recall	F1-score
AcrimSat	0.81	0.92	0.86
Aquarius	0.78	0.84	0.81
Aura	0.90	0.87	0.88
Calipso	0.78	0.76	0.77
Cloudsat	0.77	0.34	0.48
CubeSat	0.88	0.95	0.92
Debris	0.72	0.90	0.80
Jason	0.82	0.71	0.76
Sentinel-6	0.79	0.91	0.84
Terra	0.75	0.67	0.70
TRMM	0.86	0.80	0.83
Average	0.81	0.79	0.79

Table 8 demonstrates recall, precision, and F1-score of the multi-modal learning that combines DenseNet201-SVM using RGB images and End-to-End CNN using depth images. The features extracted from RGB images in DenseNet201 CNN were not able to recognize Cloudsat spacecraft with low F1 score of 8%. Therefore, the F1 score of this category was increased to 48% in multi-modal learning method. The average of recall, precision, and F1 score were 80%, 82%, and 80%, respectively as shown in Table 8.

**Table 8:** Recall, precision, and F1-score of the multi-modal DenseNet201 + End-to-End CNN.

Category	Precision	Recall	F1-score
AcrimSat	0.83	0.91	0.87
Aquarius	0.79	0.86	0.82
Aura	0.91	0.88	0.89
Calipso	0.78	0.77	0.78
Cloudsat	0.78	0.35	0.48
CubeSat	0.86	0.96	0.91
Debris	0.75	0.92	0.83
Jason	0.82	0.70	0.76
Sentinel-6	0.81	0.92	0.86
Terra	0.78	0.68	0.72
TRMM	0.86	0.82	0.84
Average	0.82	0.80	0.80

Table 9 shows recall, precision, and F1-score of the multi-modal learning that combines EfficientNetB7-SVM using RGB images and End-to-End CNN using depth images. The features extracted from RGB images in EfficientNetB7 CNN were better to recognize Cloudsat spacecraft than ones of ResNet50 and DenseNet201 but the F1 score is still low (10%). Therefore, the F1 score of this category was increased to 48% in multi-modal learning method. The average of recall, precision, and F1 score were 84%, 85%, and 83%, respectively as shown in Table 9.

**Table 9:** Recall, precision, and F1-score of the multi-modal EfficientNetB7 + End-to-End CNN.

Category	Precision	Recall	F1-score
AcrimSat	0.87	0.96	0.91
Aquarius	0.88	0.81	0.85
Aura	0.94	0.93	0.94
Calipso	0.79	0.78	0.78
Cloudsat	0.83	0.33	0.48
CubeSat	0.91	0.96	0.93
Debris	0.81	0.96	0.87
Jason	0.87	0.77	0.82
Sentinel-6	0.86	0.96	0.90
Terra	0.77	0.82	0.79
TRMM	0.85	0.92	0.88
Average	0.85	0.84	0.83

In summary, multi-modal learning method that combined a pre-trained EfficientNetB7-SVM and End-to-End CNN was found to outperform other methods that used other CNN models such as ResNet50 and DenseNet201 in terms of accuracy, precision, recall, and F1 score by 4%, 3%, 4%, and 3%, respectively. The superior performance of EfficientNetB7 was shown in all categories in Table 9.

The third experiment was done to evaluate the proposed multi-modal learning solution which combines vision transformer using RGB images and End-to-End CNN using depth images. Table 10 shows recall, precision, and F1-score of the proposed multi-modal learning. The features extracted from RGB images in vision transformer were better to recognize Cloudsat spacecraft than ones of CNN based methods. The F1 score was improved to 17%. The average of recall, precision, and F1 score were 85%, 86%, and 84%, respectively as shown in Table 10. The performance of vision transformer is shown for all categories in Table 10.

**Table 10:** Recall, precision, and F1-score of the multi-modal vision transformer + End-to-End CNN.

Category	Precision	Recall	F1-score
AcrimSat	0.82	0.97	0.89
Aquarius	0.87	0.89	0.88
Aura	0.94	0.91	0.92
Calipso	0.84	0.87	0.86
Cloudsat	0.86	0.30	0.45
CubeSat	0.84	0.98	0.91
Debris	0.83	0.95	0.88
Jason	0.88	0.81	0.85
Sentinel-6	0.87	0.94	0.90
Terra	0.78	0.80	0.79
TRMM	0.92	0.87	0.89
Average	0.86	0.85	0.84

The results of third experiment showed that the multi-modal learning method that combines a pre-trained vision transformer and End-to-End CNN outperformed all CNN based methods. First, it was found to outperform the method that used ResNet50 in terms of precision, recall, and F1 score by 5%, 6%, and 5% respectively. Similarly, it outperformed the method that used DenseNet201 in terms of precision, recall, and F1 score by 4%, 5%, and 4% respectively. On the other hand, even the method that used EfficientNetB7 has shown superior performance, vision transformer method increased each of precision, recall, and F1 score by 1%.

**Figure 5:**
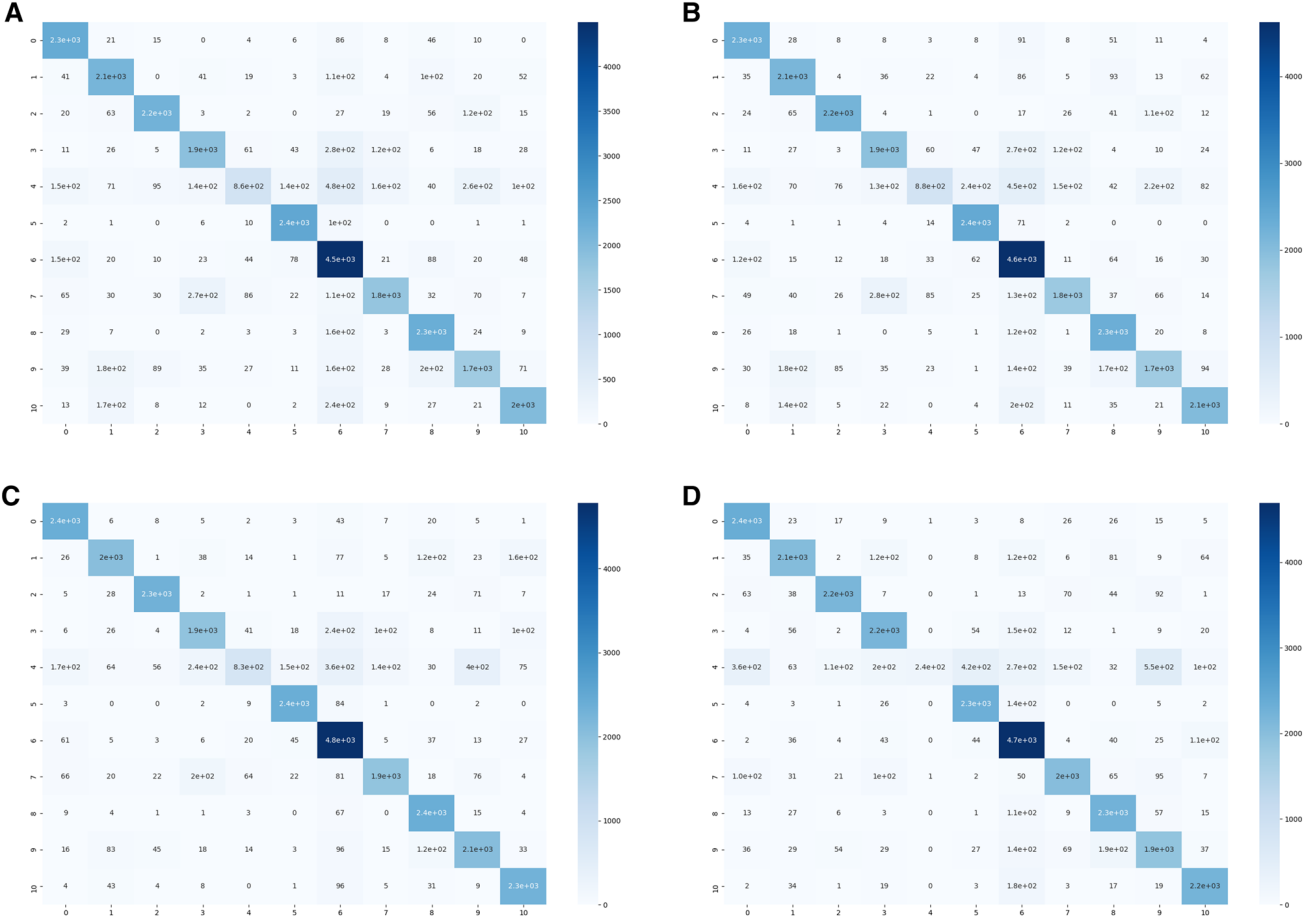
The confusion matrix of four multi-modal learning methods including (**A**) ResNet50, (**B**) DenseNet201, (**C**) EfficientNetB7, (**D**) vision transformer, combined with End-to-End CNN.

The confusion matrix of each multi-modal learning methods including CNN based methods and vision transformer was shown in Fig. 5. The multi-class classification confusion matrix illustrates the number of correct samples for each category in the main diagonal. The number of correctly predicted samples of Cloudsat category was low in all methods. Vision transformer method was able to correctly predict 2000 Jason objects and 2200 Calipso objects which is better than other CNN based methods. On the other hand, EfficientNetB7 method outperformed others by correctly predicting 2100 Terra objects and 4800 Debris objects.

The fourth experiment was conducted to compare the multi-modal learning with single-modal learning including single ResNet50 CNN + SVM, single DenseNet201 CNN + SVM, single EfficientNetB7 CNN + SVM, and single vision transformer in terms of Accuracy, precision, recall, and F1 score as shown in Table 11. It is obvious that the multi-modal learning was able to increase the correctly predicted samples by taking advantages of both RGB based and Depth-based models. The ablation study was done to validate the significance of considering both RGB and depth images instead of only RGB images. In other words, adding End-to-End CNN to learn features from depth images can increase the accuracy by 8%,7%, 5%, and 4% in multi-modal leaning methods that used ResNet50, DenseNet201, EfficientNetB7, and vision transformer, respectively. Similarly, adding End-to-End CNN for depth images increased F1 score by 10%,10%, 7%, and 6% in multi-modal leaning methods that use ResNet50, DenseNet201, EfficientNetB7, and vision transformer, respectively.

**Table 11:** Comparison between various methods in terms of accuracy, recall, precision, and F1-score. Significance values are in Bold.

Method	Accuracy	Precision	Recall	F1-score
DenseNet201—Depth	0.68	0.68	0.67	0.68
End2End CNN-Depth	0.70	0.69	0.70	0.69
ResNet50 CNN—RGB	0.72	0.76	0.70	0.69
EfficientNetB7 CNN—RGB	0.80	0.81	0.78	0.76
DenseNet201 CNN—RGB	0.74	0.77	0.72	0.70
Vision Transformer—RGB	0.81	0.83	0.80	0.78
Multi-modal (ResNet50 and End2End CNN)^2^	0.80	0.81	0.79	0.79
Multi-modal (EfficientNetB7 and End2End CNN)^2^	**0.85**	0.85	0.84	0.83
Multi-modal (DenseNet201 and End2End CNN)	0.81	0.82	0.80	0.80
Multi-modal (Vision Transformer and End2End CNN) (proposed)	**0.85**	**0.86**	**0.85**	**0.84**

In summary, multi-modal learning which is the main objective of this study has shown super performance in space domain to classify space objects into eleven categories including spacecrafts and debris. The results showed that both RGB and depth images are important to get more robust classification.

The advantages of the proposed solution are:

1. The task is formulated as image classification. It can classify the space object directly from the captured images without the need of complex localization or detection method. In other words, the proposed solution can focus the attention of model on object region of interest (ROI) inside the image and ignore irrelevant things in the background.
2. The method is robust against highly noisy images and various object sizes. Additionally, it can perform well in space missions that have various contents of backgrounds including black background, sparsely illuminated stars in the background, Earth with oceans and clouds, and object with night side or day side of Earth in the background.

**Figure 6:**
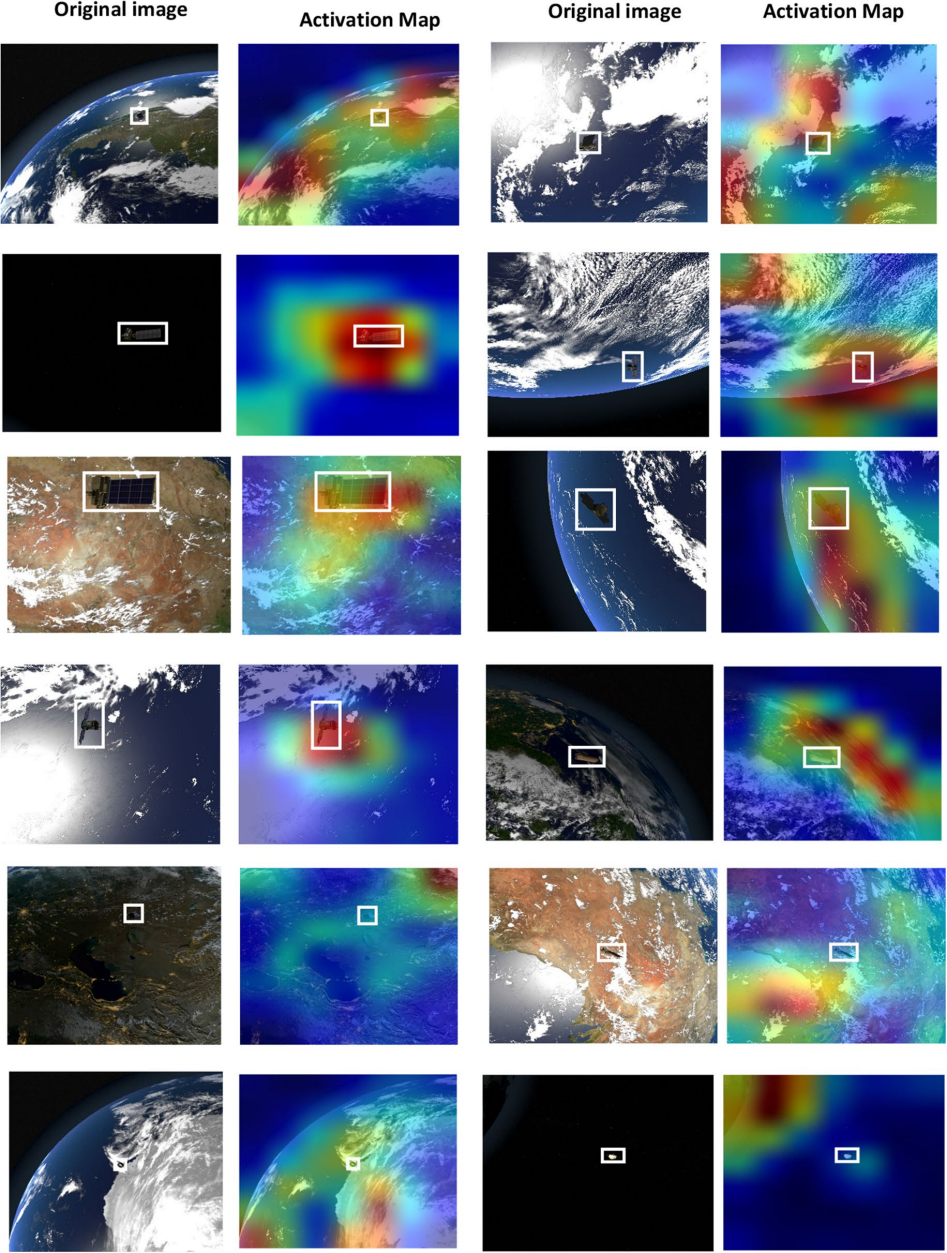
Class activation maps of a few samples that ResNet50 CNN Succeeded or failed to focus attention on space objects.

**Figure 7:**
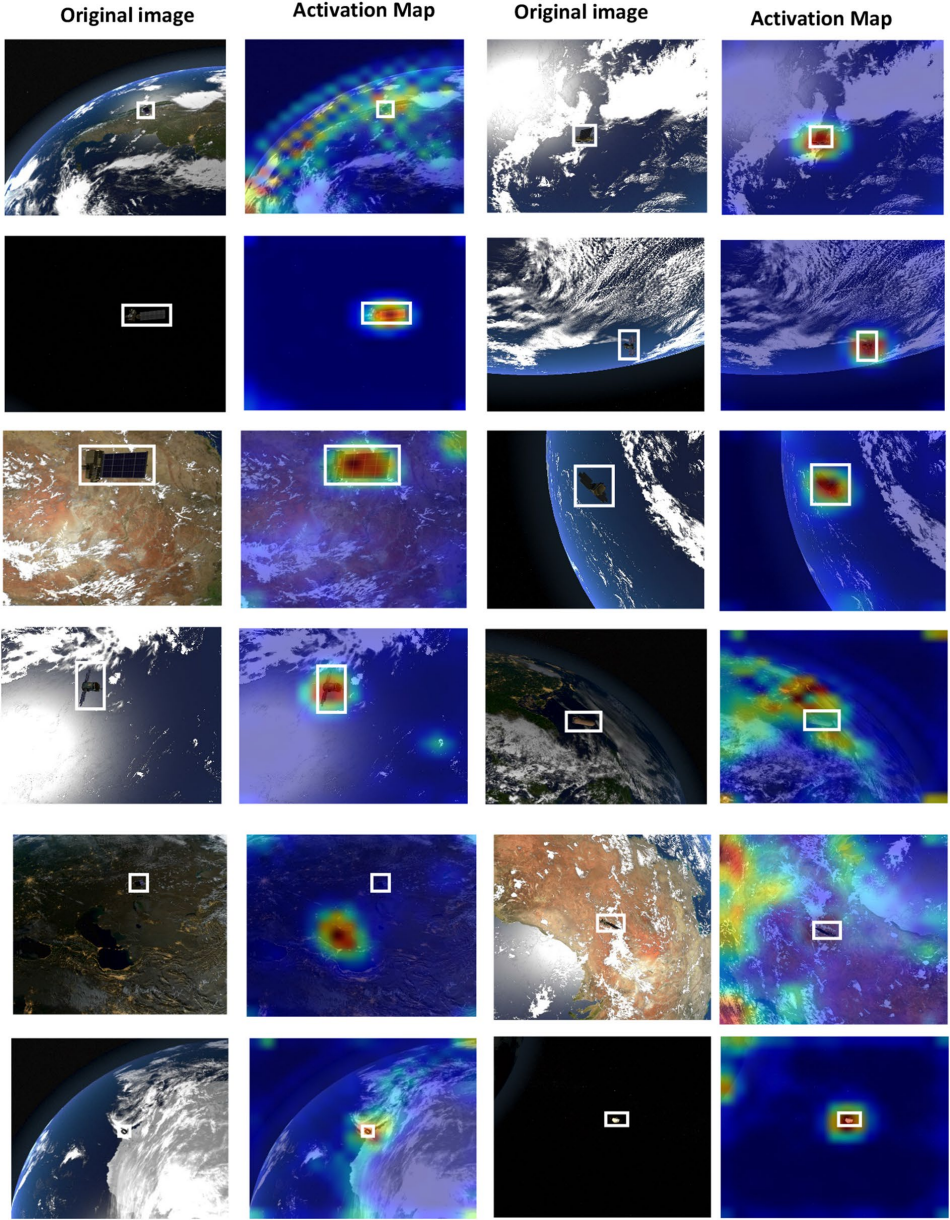
Class activation maps of a few samples that EfficientNetB7 CNN Succeeded or failed to focus attention on space objects.

**Figure 8:**
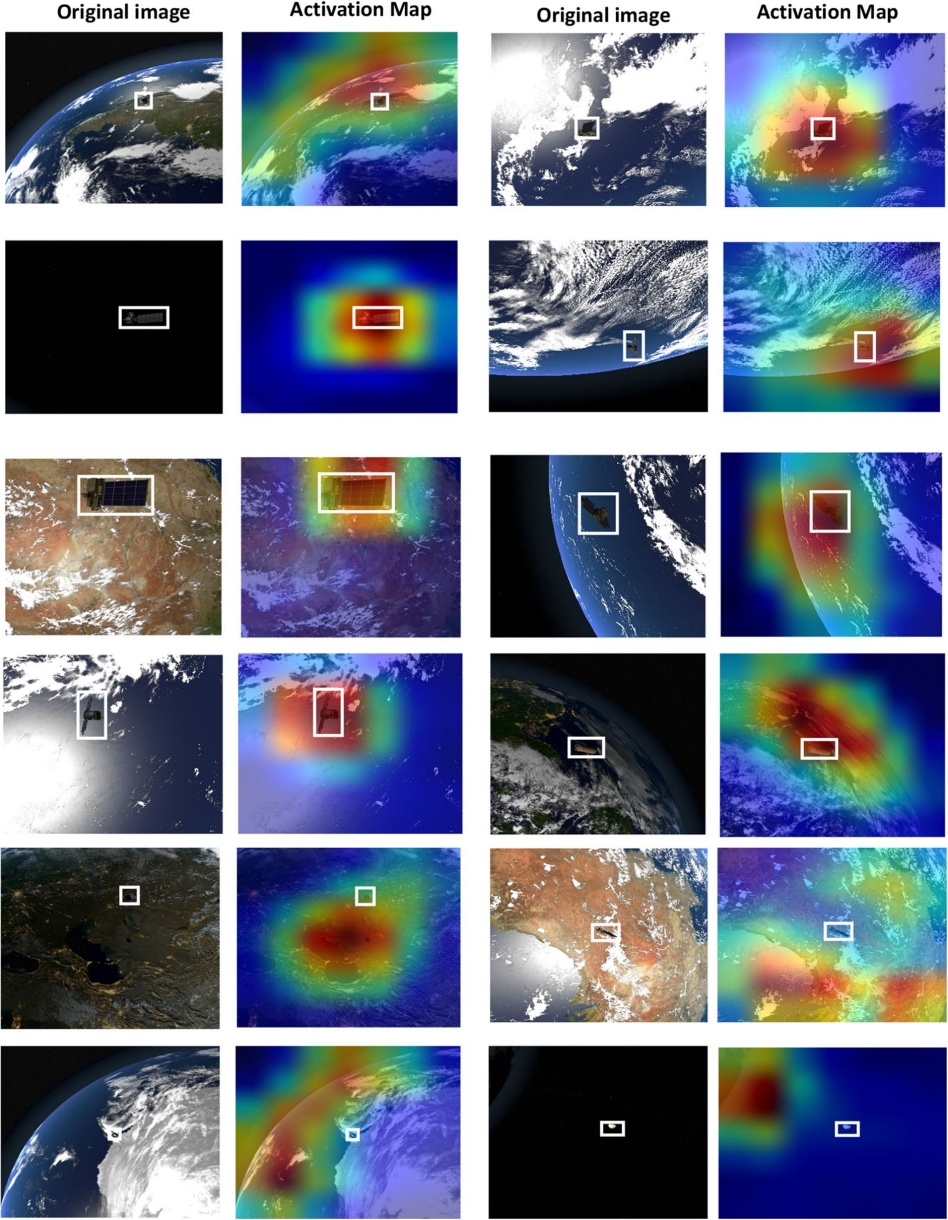
Class activation maps of a few samples that DenseNet201 CNN Succeeded and failed to focus attention on space objects.

Figures 6, 7, and 8 show class activation maps of a few samples that ResNet50, EfficientNetB7, and DenseNet201 CNNs succeeded to focus attention on space objects. On the other hand, they also show class activation maps of a few samples that CNNs failed to focus attention. It is clear that EfficientNetB7 was able to focus attention on target objects that need to be classified more than other CNNs even if the backgrounds are complex as shown in the last row. Additionally, DenseNet201 outperformed ResNet50 in several samples. The fifth row show two samples that all CNNs failed to focus their attention on the target object. The objects were surrounded by white boxes to visualize their locations clearly.

**Figure 9:**
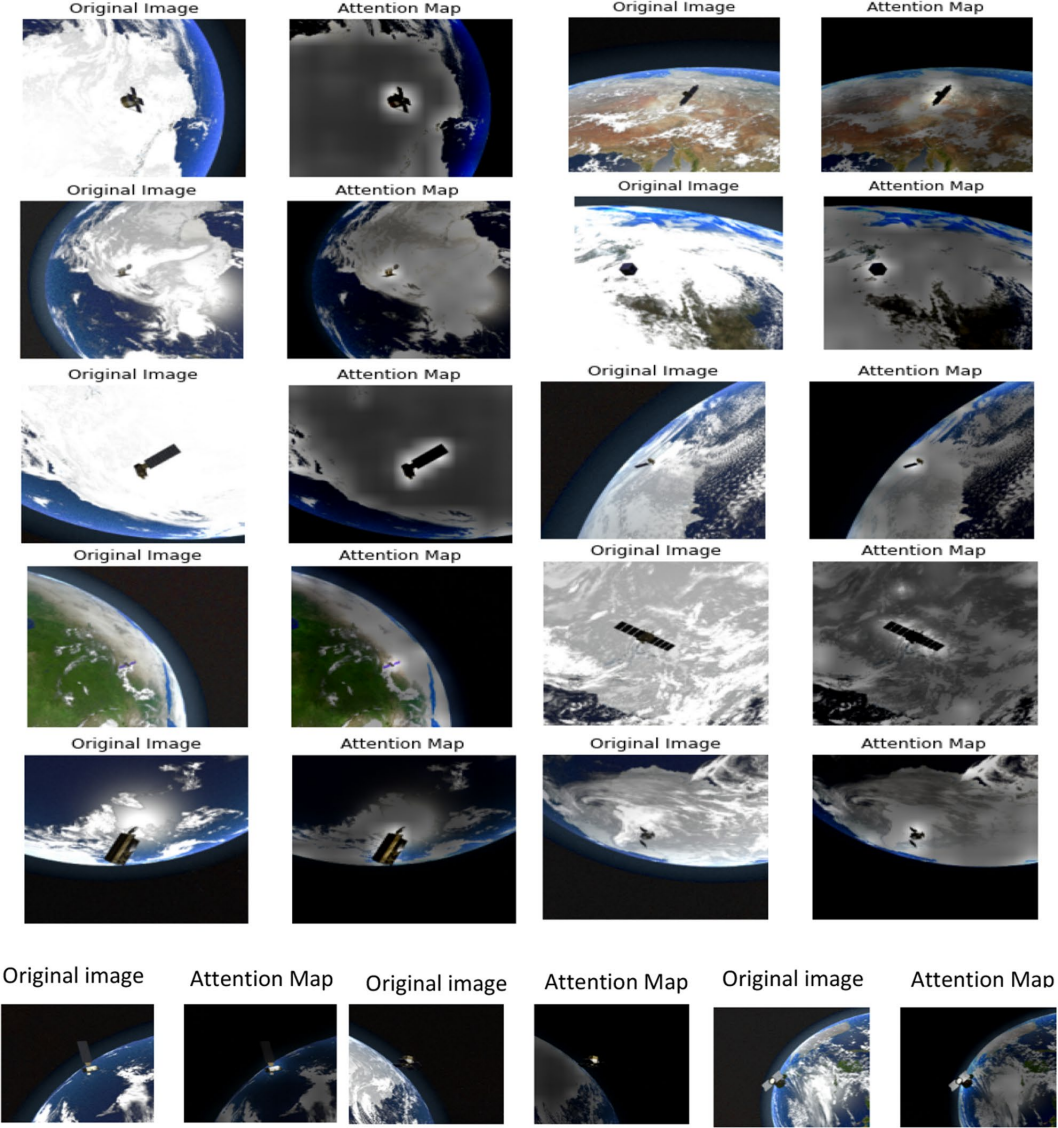
Attention maps of a few samples that vision transformer Succeeded (first five rows) and failed (last row) to focus attention on space objects.

Figure 9 shows attention maps of a few samples that vision transformer succeeded to focus attention on space objects. On the other hand, it also shows attention maps of a few samples that vision transformer failed to focus attention.

## Conclusion and future work

This paper proposed a novel solution to recognize space objects such as spacecraft and debris to enhance the performance of SSA system. A multi-modal deep learning, including a vision transformer for RGB image classification and an End-to-End CNN for depth image classification, was trained and tested with a SPARK dataset to classify eleven categories of space objects. Vision transformer was used to transfer representation from ImageNet to space images and to extract features from RGB images. The fully connected top layers of vision transformer were tuned to produce eleven probabilities of classes. At the same time, the depth images were applied to the input of End-to-End CNN to learn features and map them to eleven class probabilities. The average decision block was added to calculate the average of two sets of probabilities to make the final decision about object class. The comparison between the proposed solution and existing CNN based models such as ResNet50, EfficieneNetB7, and DenseNet201 was done. It was found that the proposed combination of RGB based vision transformer and Depth-based End-to-End CNN showed higher performance and better results in terms of accuracy (85%), precision (86%), recall (85%), and F1 score (84%). The outcome of this research work is a good feasible space recognition model that can be utilized in real task of SSA system.

The limitation in the proposed solution is inability to recognize Cloudsat category well. This category was misclassified as different categories. Additionally, the vision transformer was not able to focus attention on several samples because it was utilized only to extract features from RGB images using parameters pre-trained on ImageNet. In other words, only top layers of the transformer were tuned to fit the space images. In future, we intend to enhance the performance by fine-tuning all layers of vision transformer with SPARK images to enhance the attention maps and thus enhance the accuracy. Furthermore, this paper targets image classification task to classify the whole images applied to vision transformer. Hence, in the future, we plan to improve the recognition performance of vision transformer by formulating the problem as object detection^44^. This plays a significant role to find the object region of interest (ROI) before predicting the class which contributes to increase the accuracy.

## Data Availability

You must include a Data Availability Statement in all submitted manuscripts (at the end of the main text, before the References section); see 'Availability of materials and data' section for more information.
